# Lnc-DC promotes estrogen independent growth and tamoxifen resistance in breast cancer

**DOI:** 10.1038/s41419-021-04288-1

**Published:** 2021-10-25

**Authors:** Wan-Xin Peng, Pratirodh Koirala, Huaixiang Zhou, Jiahong Jiang, Ziqiang Zhang, Liu Yang, Yin-Yuan Mo

**Affiliations:** 1 Center of Oncology, Department of Medical Oncology, Zhejiang Provincial People’s Hospital, People’s Hospital of Hangzhou Medical College, Hangzhou, Zhejiang PR China; 2 Cancer Institute, University of Mississippi Medical Center, Jackson, MS USA; 3 Department of Biochemistry, University of Mississippi Medical Center, Jackson, MS USA; 4 Department of Pulmonary Medicine, Tongji Hospital, Tongji University, Shanghai, China; 5 Department of Pharmacology/Toxicology, University of Mississippi Medical Center, Jackson, MS USA

**Keywords:** Breast cancer, Non-coding RNAs

## Abstract

Selective estrogen receptor modulators (SERMs) such as tamoxifen have proven to be effective in the treatment of estrogen receptor (ER) positive breast cancer. However, a major obstacle for such endocrine therapy is estrogen independent growth, leading to resistance, and the underlying mechanism is not fully understood. The purpose of this study was to determine whether long non-coding RNAs (lncRNAs) are involved in regulation of estrogen independent growth and tamoxifen resistance in ER positive breast cancer. Using a CRISPR/Cas9-based SAM (synergistic activation mediator) library against a focus group of lncRNAs, we identify Lnc-DC as a candidate lncRNA. Further analysis suggests that Lnc-DC is able to reduce tamoxifen-induced apoptosis by upregulation of anti-apoptotic genes such as Bcl2 and Bcl-xL. Furthermore, Lnc-DC activates STAT3 by phosphorylation (pSTAT3^Y705^), and the activated STAT3 subsequently induces expression of cytokines which in turn activate STAT3, forming an autocrine loop. Clinically, upregulation of Lnc-DC is associated with poor prognosis. In particular, analysis of a tamoxifen-treated patient cohort indicates that Lnc-DC expression can predict the response to tamoxifen. Together, this study demonstrates a previously uncharacterized function of Lnc-DC/STAT3/cytokine axis in estrogen independent growth and tamoxifen resistance, and Lnc-DC may serve as a potential predictor for tamoxifen response.

## Introduction

Estrogen plays a critical role in breast cancer development because estrogen can stimulate cancer cell growth through estrogen receptor (ER). About 80% breast tumors express ER and thus, specific targeting of the estrogen signaling pathway provides an effective way to treat ER positive breast cancer. Several clinical drugs currently available target either estrogen synthesis such as aromatase inhibitors or ER such as selective estrogen receptor modulators (SERMs) (e.g., tamoxifen). However, over 30% of these ER positive tumors fail to respond to tamoxifen therapy due to intrinsic resistance [1]; moreover, those that initially respond to tamoxifen frequently develop resistance to the treatment (acquired resistance) [2]. Although it is believed that this likely results from the activation of estrogen independent signaling, the precise molecular mechanism is not fully understood.

Previous studies indicate that multiple factors are attributed to tamoxifen resistance, including AKT [2], mutations and SNPs in ER [3, 4], crosstalk among growth factors [5, 6], post-transcriptional regulation by microRNAs [7]. However, less is known whether and how long non-coding RNAs (lncRNAs) impact estrogen independent growth and tamoxifen resistance. LncRNAs are arbitrarily defined as a group of non-coding RNAs with molecular weight of > 200 nt in length [8, 9] and they may play a critical role in regulation of normal cellular functions and disease processes. In particular, the dysregulation of lncRNA expression is often associated with a variety of human diseases including cancer. For example, a number of lncRNAs have been implicated in cancer initiation, progression, and metastasis as well as stem cell maintenance [10–15]. This may have to do with their ability to interact with DNA, RNA, or protein to regulate gene expression through which lncRNAs may serve as (1) signals for transcription; (2) decoys to titrate transcription factors; (3) guides for chromatin-modifying enzymes to be recruited to target genes, and (4) scaffolds to bring together multiple proteins to form functional ribonucleoprotein complexes [16–19]. Therefore, our goal of this study was to explore whether lncRNAs can regulate genes involved in estrogen independent growth and tamoxifen resistance.

Specifically, we explored the utility of CRISPR/Cas9 gene editing system [20] to identify lncRNAs involved in estrogen independency and tamoxifen resistance. We used a synergistic mediation activator (SAM) for transcription activation [21] to screen a SAM library against a focus group of lncRNAs. We showed that upregulation of Lnc-DC induces estrogen independent growth and tamoxifen resistance through regulation of the STAT3 and cytokine loop.

## Materials and methods

### Reagents

Primary antibodies were purchased from commercial sources: pSTAT3^Y705^, STAT3, and SHP-1 were from Cell Signaling (Danvers, MA, USA); GAPDH and Tubulin were from Protein Tech (Chicago, IL, USA). Secondary antibodies conjugated with IRDye 800CW or IRDye 680 were purchased from LI-COR Biosciences (Lincoln, NE, USA). PCR primers were obtained from IDT (Coralville, IA, USA). STAT3 siRNAs and control siRNA were purchased from Thermo Fisher Scientific (Waltham, MA, USA). Cytokine Array kit was obtained from R&D system Inc. (Minneapolis, MN).

### Cell culture

MCF-7, T47D, and 293 T cells were obtained from ATCC (Manassas, VA, USA). MCF-7 and T47D cells were grown in RPMI 1640 (Thermo Fisher Scientific) with 10% FBS (Sigma-Aldrich, St. Louis, MO, USA) and 2 mM glutamine (Thermo Fisher Scientific). For estrogen deprivation experiments, cells were grown and maintained in phenol-red-free RPMI-1640 medium supplemented with 5% charcoal-stripped FBS (Thermo Fisher Scientific), 2 mM L-glutamine, 100 U/ml penicillin and 100 μg/ml streptomycin. 293 T cells were grown in DMEM with 10% FBS supplemented with 2 mM L-glutamine, 100 U/ml penicillin, and 100 μg/ml streptomycin. The cells were authenticated by DDC Medical (http://www.ddcmedical.com) using the short tandem repeat profiling method. All cells were incubated at 37 °C with 5% CO_2_ in the humidified chamber.

### Plasmid constructs

To knock out Lnc-DC, we adopted the dual gRNA approach with CRISPR/Cpf1 and Lnc-DC dual gRNAs were cloned using the approach as previously described [22]. To clone single Lnc-DC gRNAs in SAM system, we digested the modified lenti sgRNA (MS2)_zeo backbone vector (see below) with BsmB1 and then ligated with synthetic gRNA oligos (Supplementary Table 1) which were compatible with the linearized vector. Briefly, two complementary oligos were heated at 95 °C and then annealed gradually to room temperature, and finally ligated into lenti sgRNA (MS2)_zeo vector in the presence of T4 DNA ligase (New England Biolabs). To clone Lnc-DC into S1m vector [23], we first amplified Lnc-DC with primers Lnc-DC-S1m-R1-5.1 and Lnc-DC-S1m-BamH1-3.1 (Supplementary Table 1) and then cloned into the vector at EcoR1 and BamH1 site. The high fidelity DNA polymerase Phusion enzyme (New England BioLabs) was used for PCR. All PCR products were verified by DNA sequencing.

### Modification of lenti sgRNA (MS2)_zeo backbone vector

To construct SAM library, we modified lenti sgRNA(MS2)_zeo backbone vector (Addgene Plasmid #61427) by replacing the Nhe I-BamH I fragment with a gBlock (IDT) fragment (Supplementary Table 1) such that PCR products derived from the mixed oligo pool can be cloned into this vector by Gibson assembly method [24].

### Construction of SAM library

SAM gRNA library was constructed by designing gRNAs against ~1 kb from promoter regions of 241 different lncRNAs. Five gRNAs were designed for each lncRNAs using Chop-Chop program [25]. Oligonucleotides (113 nt in length) carrying each gRNA were synthesized as a mixed pool (CustomArray Inc) (Supplementary Table 2) and then amplified by PCR using primers SAM gRNA-5.1 and SAM gRNA-3.1 (Supplementary Table 1) and finally cloned into the modified lenti sgRNA(MS2)_zeo vector by Gibson assembly method.

### Library screening

MCF-7 cells were first infected with lenti MS2-P65-HSF1_Hygro (Addgene Plasmid #61426) and lenti dCAS-VP64_Blast (Addgene Plasmid #61425) and then selected with hygromycin and blasticidin. Next, SAM library was introduced into these cells by infection, followed by zeocin selection. The cells were cultured in E2 free medium for 28 days during which the medium was replaced with fresh E2 free medium every other 2 days. After that, the cells were harvested for lncRNA profiling.

### LncRNA profiling

Since number of lncRNAs in this SAM gRNA library was relatively small, we profiled lncRNA expression using PCR arrays consisting of primer sets derived from each of these lncRNAs included in the library. Total RNA from cells was isolated using Direct-zol™ RNA MiniPrep kit (Zymo Research, Irvine, CA, USA). Reverse transcription was carried out by using RevertAid Reverse Transcriptase (Thermo Fisher Scientific) and random primer mix (New England BioLabs). The expression of lncRNAs was detected by quantitative PCR (qPCR) using SYBR Green method. Analysis of qRT-PCR was performed as described previously [26]. For comparison, we included the cells carrying the same SAM library before estrogen deprivation.

### Transfection

Cells were transfected with plasmid DNA using DNAfectin (Applied Biological Materials) or with siRNAs using RNAfectin reagent (Applied Biological Materials) following the manufacturer’s protocol.

### Lentivirus infection

Lentivirus was packaged in 293 T cells using the third generation of packaging system and the virus containing culture medium was collected 48 h after transfection and spun for 10 min at 3,000 r.p.m. Lentivirus infection was carried out six-well plates by mixing 500 μl virus supernatant + 500 μl medium containing 8 μg polybrene.

### MTT assay

Cell proliferation was analyzed using MTT assays as described previously [27]. Cells were seeded in 96-well plates at a density of 3.5 × 10^3^ cells per well and grown for 3 days before adding MTT.

### Colony formation assay

For the colony formation assay, cells were seeded in six-well plates. After two weeks of culture, the cells were fixed with 4% PFA (Sigma) and then stained with crystal violet (0.5%). The number of colonies were countered for 5 representative fields. The experiments were repeated 3 times. Relative colony formation was calculated as compared to vector controls as 100%.

### Knockout of Lnc-DC by CRISPR/Cpf1

We knocked out Lnc-DC by CRISPR/Cpf1 system [28] using the dual gRNA approach, as described previously [22]. In brief, dual gRNA targeting the outside regions of Lnc-DC gene was designed using Benchling (https://benchling.com) and primer sequences were listed in Supplementary Table 1. Oligonucleotides carrying dual gRNA targeting Lnc-DC were synthesized (IDT Inc.) and then cloned into pY108 at BsmB1 site (Addgene, Watertown, MA, USA). We introduced empty vector or dual gRNA expression vector into MCF-7 and T47D cells by infection. Three days later, the infected cells were subject to puromycin (1 μg/ml) selection for 5 days. Individual puromycin resistant colonies were picked up manually and then expanded in 12-well plates. Initial identification of KO clones was carried out by genomic PCR. The clones were further verified by qRT-PCR.

### RNA immunoprecipitation

RNA immunoprecipitation was performed using Magna RIP RNA-Binding Protein Immunoprecipitation Kit (Millipore, Billerica, MA) using STAT3 antibody according to the manufacturer’s protocol. After the RNA was recovered from protein A + G beads, qRT-PCR was performed to detect Lnc-DC using primers Lnc-DC-RT-5.1 and Lnc-DC-RT-3.1 (Supplementary Table 1).

### Chromatin immunoprecipitation

Chromatin immunoprecipitation (ChIP) assays were performed using EZ-ChIP^TM^ (Millipore, Billerica, MA, USA) according to the manufacturer’s protocol. Briefly, cells were first fixed with 1% formaldehyde to generate DNA–protein cross-links. Then, cells were lysed and sonicated to chromatin fragments of ~200–300 bp and cell lysates were immunoprecipitated with STAT3 antibody. After immunoprecipitation, genomic DNA was isolated and bound protein was digested with proteinase K. PCR was performed using primers listed in Supplementary Table 1. IgG and Ku80 antibody were used as negative antibody controls.

### In vivo S1m precipitation

To detect in vivo interaction between Lnc-DC and STAT3, we constructed S1m-tagged Lnc-DC expression vector, and then transfected it into MCF-7 cells. The cells were harvested 48 h after transfection and then lysed in 1 ml lysis buffer containing protein inhibitors and RNase inhibitor. The supernatant was collected after centrifugation for 20 min at 4 °C and then incubated with streptavidin beads (MilliporeSigma) for 30 min at 4 °C to remove the background. The pre-cleaned cell lysate was transferred to a new tube and 1/10 of the lysate was saved as protein input. The remaining lysate was incubated with streptavidin beads for 4 h at 4 °C before washing 5 times with ice-cold PBS. Finally, the pellet was dissolved in 30 µl 2xSDS sample buffer, followed by SDS-PAGE and Western blot with STAT3 antibody.

### Western blot

The cells were treated as per requirement, and protein was extracted form cells. Concentration of protein was estimated by Bradford method [29]. The protein sample was run in SDS-polyacrylamide gel and probed with antibody of interest. Signals were detected in the Odyssey system (LI-COR Biosciences).

### Cytokine array

To identify the cytokines secreted in conditioned medium from the cells, we used the cytokine array kit carrying antibodies against 102 cytokines from R&D systems (Proteome Profiler™ Human XL Cytokine Array Kit), and experiments were performed according to the manufacturer’s protocol using Odyssey imaging system (Li-Cor).

### Animal work

Female nude (nu/nu) mice (4–5 weeks old) were purchased from Charles River (Wilmington, MA, USA). All animal studies were conducted in accordance with the NIH animal use guidelines and a protocol was approved by the UMMC Animal Care Committee. MCF-7 cells carrying SAM library before or after selection at the exponential stage were harvested at exponential stage of growth and mixed with 50% matrigel (BD Biosciences). One million cells/spot were injected to mammary fat pads of female nude mice. Tumor growth was monitored and size of tumor was measured every other day after 7 day of cell injection.

### Statistical analysis

Statistical analysis of data was performed using the Student’s *t*-test or one way ANOVA with the help of Prism software version 6. A value of *P* < 0.05 was considered statistically significant.

## Result

### Lnc-DC is identified as a potential lncRNA involved in estrogen independent growth; its upregulation is associated with breast cancer patient survival and tamoxifen response

We adopted a CRISPR/Cas9-based synergistic mediation activator (SAM) system using MS2 bacteriophage coat proteins combined with p65 and HSF1 because it has been shown to greatly enhance the transcription activation [21]. Unlike ectopic expression of a gene of interest, this Cas9-based SAM system is able to activate transcription of endogenous genes without impacting other events such as gene splicing. Thus, it is expected to better recapitulate the effect on gene function. Toward this end, we generated gRNAs targeting the putative promoter against 241 lncRNAs (5 gRNA per lncRNA) (Supplementary Table 1); these lncRNAs were selected primarily based on two sources (www.lncrandb.org and http://www.cuilab.cn/lncrnadisease) and they are curated from the literature.

**Fig. 1:**
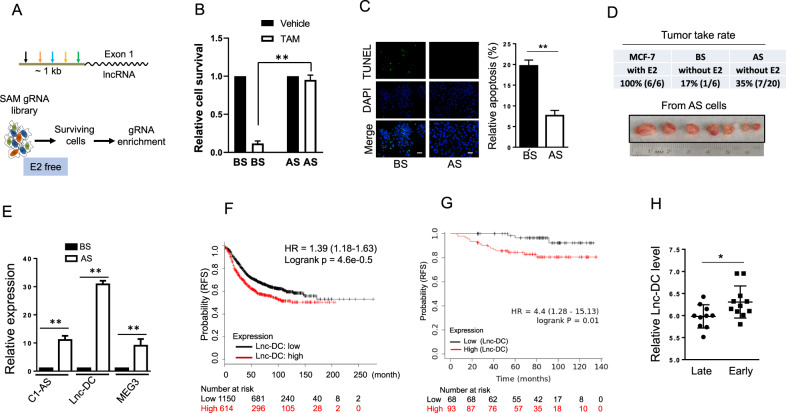
Identification of Lnc-DC as a potential lncRNA involved in estrogen independency and tamoxifen resistance. **A** Design for SAM gRNAs and procedure for SAM library screen. **B** Responses of cells before selection (BS) and after selection (AS) to tamoxifen. The cells were treated with 10 μM tamoxifen for 3 days before relative cell survival was determined by MTT assay. **C** Suppression of tamoxifen-induced apoptosis in AS cells, as determined by TUNEL assay. Relative apoptosis was shown on the right. **D** AS cells result in a higher tumor rate without E2 than that of BS cells without E2. **E** Expression of three lncRNAs (C1qTNF2B-AS1, Lnc-DC, and MEG3) in AS cells vs BS cells, as detected by qRT-PCR. **F** Upregulation of Lnc-DC (WFDC21P) is associated with poor relapse free survival (RFS) in 1764 breast cancer cases, using the kmplot program (www.kmplot.com) with auto select best cutoff setting. **G** Upregulation of Lnc-DC is associated with poor RFS in 161 breast cancer cases in response to tamoxifen treatment, using the same kmplot program with auto select best cutoff setting. Cohort: ER positive breast cancer patients with systemic tamoxifen. **H** Expression of Lnc-DC in tamoxifen treated patients with early and late relapse based on analysis of GSE16391 (see Fig. S3). Values are mean ± SD (*n* = 3 except for 1E–G). **, *p* < 0.01; *, *p* < 0.05; bar, 10 µm.

To determine the involvement of lncRNAs in estrogen-independent growth, we performed gain of function screens. Using the selection procedure outlined in Figs. 1A and S1, we found that after 28 days of estrogen deprivation, there were very few surviving cells for vector control whereas there were more surviving cells for the SAM gRNA library, suggesting that SAM gRNAs which can promote cell survival in E2 free medium were enriched. Since estrogen independency often leads to tamoxifen resistance, we asked whether this procedure affects the response to tamoxifen in these cells. As expected, cells after selection (AS) were more resistant to tamoxifen than before selection (BS) cells (Fig. 1B). Consistent with these findings, TUNEL assays revealed that AS cells revealed a much lower apoptotic rate (~8%) than BS cells (~20%) in response to tamoxifen (Fig. 1C).

Next, we injected BS cells or AS cells into mammary fat pads of female nude mice to determine whether they can grow into tumor without supplement of estrogen. As expected, the parental MCF-7 supplemented with E2 (estradiol pellet) resulted in 100% tumor take rate (Fig. 1D). Tumor take rate for BS cells without E2 was 17%; in contrast, tumor take rate for AS cells without E2 was 35% (Fig. 1D), further suggesting the involvement of lncRNAs in promoting estrogen independent growth.

To identify which SAM gRNAs are enriched by this selection, we adopted a two-step approach. The first step was to perform lncRNA profiling by qRT-PCR arrays against each of lncRNAs in this library. The second step was to detect the relative abundance of SAM gRNAs by qRT-PCR using gRNA specific primer sets, focusing on those enriched by estrogen deprivation. The lncRNA profiling identified that three lncRNAs (C1qTNF9B-AS1, Lnc-DC and MEG3) were upregulated (Fig. 1E) after selection (AS) as compared to before selection (BS).

Based on the profiling results, we asked whether any of these lncRNAs has any clinical relevance. Searching kmplot (http://kmplot.com/analysis/index.php?p=service&cancer=breast) breast cancer gene chip database [30] revealed that C1qTNF9B-AS1 was not in the database; both Lnc-DC and MEG3 were in the database. Of interest, upregulation of Lnc-DC was associated with poor relapse free survival (RFS) with Logrank 4.6e-0.5 and HR = 1.39 among 1,764 breast cancer cases (Fig. 1F). By contrast, upregulation of MEG3 was associated with better patient survival (Fig. S2A). More importantly, Lnc-DC was able to predict the response to tamoxifen therapy. In a cohort consisting of 161 cases that received systematic tamoxifen treatment, we found that Lnc-DC expression was able to differentiate good responders from poor responders. For instance, high Lnc-DC level was associated with poor RFS with Logrank 0.01 and HR = 4.4 (Fig. 1G). However, MEG3 expression was not associated with the response to tamoxifen treatment (Fig. S2B). In addition, analysis of GEO database (GSE#16391) revealed that patients with a high level of Lnc-DC had a shorter time for relapse (early relapse). In this regard, we analyzed RFS for each patient and calculated the mean RFS (36.39 ± 3.88 months) (Fig. S3). We used this mean value as a divider to have two groups, i.e., early relapse group (< 36.39 months) and late relapse group (> 36.39 months). This analysis also supported the notion that patients with a high level of Lnc-DC are poor responders for tamoxifen (Fig. 1H), suggesting the clinical importance of Lnc-DC. Therefore, we further characterized Lnc-DC for the following experiments.

### Lnc-DC promotes tamoxifen resistance

**Fig. 2:**
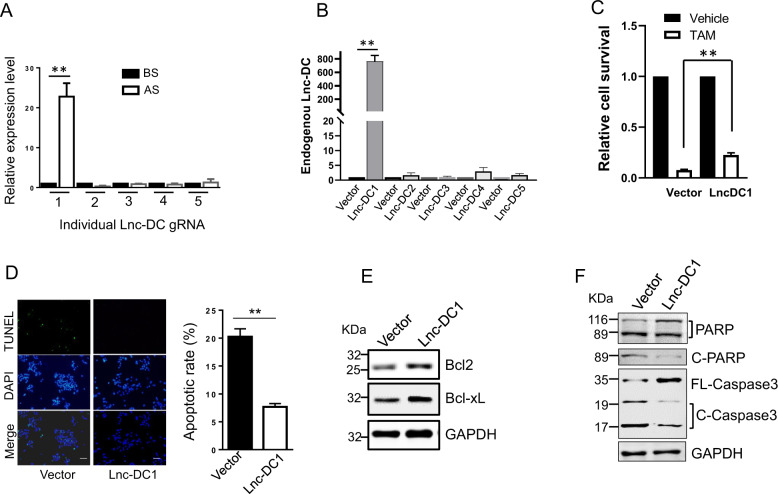
Lnc-DC promotes tamoxifen resistance. **A** Lnc-DC gRNA 1 (Lnc-DC1) is only one of 5 gRNAs enriched after estrogen deprivation selection using gRNA specific primers. **B** Lnc-DC1 causes upregulation of the endogenous Lnc-DC. Each of 5 Lnc-DC gRNAs was individually introduced into MCF-7 SAM cells and their expression was determined by qRT-PCR using Lnc-DC specific primers Lnc-DC-RT-5.1 and Lnc-DC-RT-3.1. **C** Lnc-DC1 expressing cells are more resistant to tamoxifen (TAM). The cells infected with vector or Lnc-DC1 expression lentivirus were treated with 10 μM tamoxifen for 3 days before relative cell survival was determined by MTT assay. **D** Suppression of tamoxifen-induced apoptosis in Lnc-DC1 expressing cells, as determined by TUNEL assay. **E** Upregulation of Bcl2 and Bcl-xL in Lnc-DC1 expressing cells, as detected by Western blot. **F** Lnc-DC suppresses the cleavage of PARP and Caspas3. Values are mean ± SD (*n* = 3). **p < 0.01; bar, 10 µm.

To confirm the contribution of Lnc-DC gRNAs to upregulation of the Lnc-DC expression, we designed primers from the gRNA region and the scaffold region such that we would be able to detect the exogenous Lnc-DC gRNA levels. Although 5 different Lnc-DC gRNAs were included in this SAM library, only Lnc-DC gRNA 1 (Lnc-DC1) was highly enriched after selection (Fig. 2A); other 4 gRNAs were mostly reduced after selection. Next, we constructed 5 Lnc-DC gRNAs individually and then introduced each of them into cells separately to determine their effect on the endogenous Lnc-DC expression. Indeed, we found that only Lnc-DC1 was able to activate the endogenous Lnc-DC expression (Fig. 2B), suggesting that although not all gRNAs are able to induce the endogenous gene expression, those gRNAs that do can be enriched by means of selection such as estrogen deprivation.

Next, we determined the contribution of Lnc-DC to tamoxifen resistance. We treated the Lnc-DC1 cells and vector control cells with tamoxifen. As shown in Fig. 2C, Lnc-DC1 cells were 3-fold more resistant to tamoxifen than vector control. Similarly, Lnc-DC1 resulted in less apoptotic rate than vector control (Fig. 2D). Moreover, Lnc-DC1 cells revealed an increased level of Bcl2 and Bcl-xL (Fig. 2E), which may be in part responsible for the observed resistance to tamoxifen. Lnc-DC also decreased the levels of cleaved PARP and Caspase 3 (Fig. 2F).

**Fig. 3:**
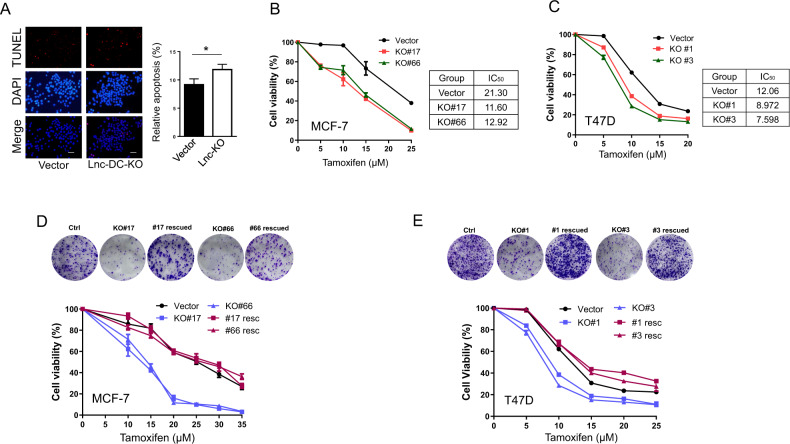
Determination of Lnc-DC-mediated tamoxifen resistance by Lnc-DC KO and rescue assays. **A** Lnc-DC KO increases tamoxifen-induced apoptosis, as detected by TUNEL assays. **B**, **C** Lnc-DC KO cells (MCF-7 and T47D) have a low IC50 in response to tamoxifen, as determined by colony formation assays. The cells were treated with indicated concentrations of tamoxifen, and colonies were countered 2 weeks after the treatment. **D**, **E** Re-expression of Lnc-DC in KO cell restores the response to tamoxifen as vector control in both MCF-7 and T47D cells, as determined by colony formation assays. Values are mean ± SD (*n* = 3). *p < 0.05; bar, 10 µm.

To better define the role of Lnc-DC in tamoxifen resistance, we generated Lnc-DC knockout (KO). It is evident that Lnc-DC KO cells were more sensitive to tamoxifen as determined by TUNEL assay (Fig. 3A). Then, we picked two single Lnc-DC KO clones and at the same time, we included another ER positive breast cancer cell line T47D (Fig. S4). Colony formation assays revealed that in MCF-7 cells, both Lnc-DC KO clones were more sensitive to tamoxifen (Fig. 3B). For instance, IC50 for MCF-7 Lnc-DC KO was 11.6 (KO#17) and 12.92 (KO#66) as compared to vector control (IC50 of 21.3). Similarly, Lnc-DC KO T47D cells were also more sensitive to tamoxifen as compared to vector control (Fig. 3C). To further determine the role of Lnc-DC in tamoxifen resistance, we performed rescue experiments, i.e., we re-expressed Lnc-DC in the KO cells. In both MCF-7 and T47D cells, we found that Lnc-DC KO increased sensitivity to tamoxifen; by contrast, re-expression of Lnc-DC in the KO cells restored the tamoxifen response as vector control (Fig. 3D, E). Together, these results support the role of Lnc-DC in tamoxifen resistance.

### Lnc-DC promotes phosphorylation of STAT3

**Fig. 4:**
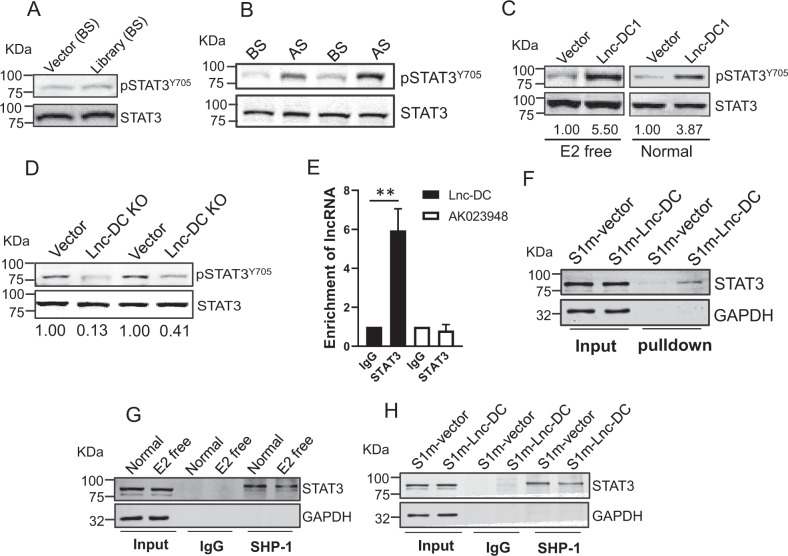
Lnc-DC promotes STAT3 activity by its interaction with STAT3. **A** There is no difference between vector control and library cells in the level of pSTAT3^Y705^ before selection. **B** AS cells express a higher level of pSTAT3^Y705^ than BS cells. **C** Lnc-DC1 upregulates the level of pSTAT3^Y705^ as compared to vector control in either E2 free or normal medium. **D** Lnc-DC KO decreases the pSTAT3^Y705^ level. **E** Lnc-DC interacts with STAT3, as determined by RNA immunoprecipitation using STAT3 antibody. AK023948 serves as a negative control. Values are mean ± SD (*n* = 3). **p < 0.02. **F** Interaction of Lnc-DC with STAT3 as determined by in vivo precipitation assay. MCF-7 cells were infected with S1m- Lnc-DC or S1m vector control. Two days later, cellular extracts were prepared for streptavidin precipitation, followed by western blot. **G** Estrogen deprivation reduces the interaction between STAT3 and SHP-1, as determined by co-immunoprecipitation with SHP-1 antibody. MCF-7 cells were cultured in E2 free medium for 7 days before harvesting for co-immunoprecipitation. **H** Lnc-DC also reduces the interaction between STAT3 and SHP-1, as determined by co-immunoprecipitation with SHP-1 antibody. MCF-7 cells were first infected with S1m-Lnc-DC, and 2 days later, the cells were harvested for co-immunoprecipitation with SHP-1 antibody.

To determine how Lnc-DC promotes tamoxifen resistance, we examined phosphorylation of STAT3 in these cells because Lnc-DC has been previously shown to regulate phosphorylation of STAT3 at tyrosine 705 in dendritic cells [31]. Moreover, phosphorylation of STAT3 has been implicated in upregulation of anti-apoptotic signals, leading to tamoxifen resistance [32]. We first examined the phosphorylation of STAT3 in the cells carrying the SAM gRNA library before selection and found that there were no difference between vector and library cells (Fig. 4A). However, after selection, the library cells displayed a higher level of phosphorylated STAT3 than the BS cells (Fig. 4B). To determine the role of Lnc-DC in activation of STAT3, we overexpressed Lnc-DC and found that Lnc-DC1 was able to increase the level of pSTAT3^Y705^ in both normal and E2 free medium (Fig. 4C). Furthermore, Lnc-DC KO caused a decrease in the level of pSTAT3^Y705^ (Fig. 4D). RNA immunoprecipitation assay with STAT3 antibody confirmed that Lnc-DC interacted with STAT3 (Fig. 4E). For instance, there was ~ a 6-fold enrichment of Lnc-DC by STAT3 antibody, whereas there was no such enrichment for AK023948, a negative control. To further determine the interaction between Lnc-DC and STAT3, we performed in vivo RNA precipitation. In this case, we first tagged Lnc-DC with S1m which mimics the function of biotin with a high affinity with streptavidin [33]. We have successfully used this approach to detect the interaction of DANCR with its binding partner [23]. As shown in Fig. 4F, we detected a STAT3 band in the S1m-Lnc-DC precipitate. These results strongly support that the interaction between Lnc-DC and STAT3 is specific.

To determine how Lnc-DC impacts the STAT3 activity, we found that estrogen deprivation decreased the interaction of STAT3 with SHP-1 (Fig. 4G), a phosphatase which has been implicated in regulation of STAT3 activity [34]. Estrogen deprivation or Lnc-DC expression had no effect on SHP-1 expression (Fig. S5). Importantly, Lnc-DC caused a reduction of the interaction between SHP-1 and STAT3 (Fig. 4H), which may explain in part the increased STAT3 activity when the level of Lnc-DC is elevated.

### Activation of STAT3 by cytokines secreted from tamoxifen resistant AS cells

**Fig. 5:**
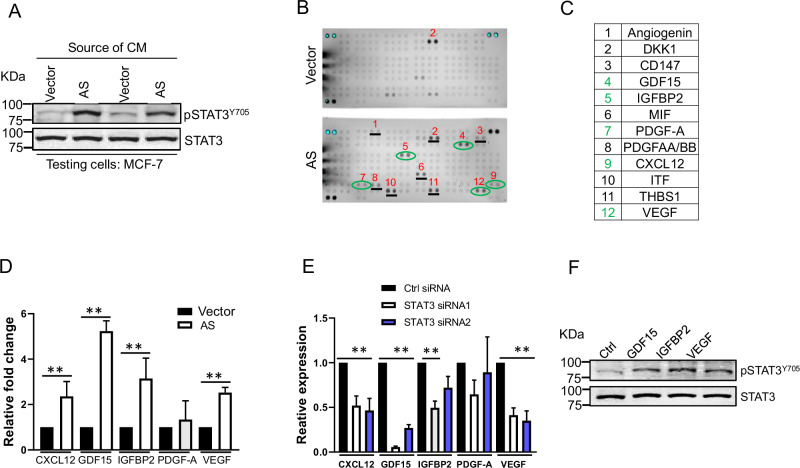
STAT3-mediated production of cytokines. **A** Conditioned medium from AS cell culture increases level of pSTAT3^Y705^. The cells were grown in E2 free medium for 3 days before harvesting conditioned medium which was then added to MCF-7 culture. The cells were further cultured for 24 h before western blot. **B** Detection of different cytokines secreted in vector control cell and AS cell culture. The cells were cultured in E2 free medium for 2 days before the conditioned medium was harvested for cytokine array analysis. **C** Upregulation of 12 cytokines in AS cells with over a 2-fold as compared to vector control cells. **D** Upregulation of 5 cytokines at the mRNA level in AS cells as compared to vector control, as detected by qRT-PCR. The cells were prepared as in (**A**) before harvesting for RNA extraction. **E**, STAT3 siRNAs downregulate cytokine expression in MCF-7 cells, as detected by qRT-PCR. **F** Cytokines GDF15, IGFBP2 and VEGF stimulate STAT3 activity. MCF-7 cells were treated with GDF15, IGFBP2, and VEGF at 1 nM for 1 h before harvesting for western blot. Values are mean ± SD (*n* = 3). **, *p* < 0.01.

It is well known that STAT3 is activated by phosphorylation at Tyr705, which induces dimerization, nuclear translocation, and expression of downstream target genes including cytokines [35]. Since these tamoxifen resistant cells express a high level of pSTAT3^Y705^, we asked whether these cells produce cytokines that are secreted into the medium. Therefore, we collected the conditioned medium from the vector control and AS cells, respectively, and then treated the parental MCF-7 cells that were initially grown in normal medium. The clear activation of pSTAT3^Y705^ was observed in the conditioned medium from the AS cells as compared to vector control (Fig. 5A), suggesting that cytokines are secreted into medium and are capable of activating STAT3 in neighboring cells.

To determine what cytokines are secreted from the AS cell culture, we performed cytokine analysis from the conditioned medium by cytokine arrays that carrying antibodies against 102 cytokines. Indeed, a group of 11 cytokines were detected to be higher in medium derived from AS cells than in vector control cells (Fig. 5B). The qRT-PCR analysis verified 5 of them (GDF15, IGFBP-2 PDFG-A, CXCL12, and VEGF) (Fig. 5C in green) and they were also upregulated in AS cells as compared to vector control (Fig. 5D). To determine whether STAT3 can regulate these cytokine genes, we knocked down STAT3 in MCF-7 cells by 2 different siRNAs (Fig. S6). The qRT-PCR analysis indicated that four of those 5 cytokine genes were substantially downregulated in STAT3 KD cells (Fig. 5E). Especially for GDF15, the suppression was the most obvious. Finally, treatment of MCF-7 cells with GDF15, IGFBP-2, and VEGF enhanced the phosphorylation of pSTAT3^Y705^ as compared to control (Fig. 5F). These results suggest that there is a positive feedback loop involving STAT3 and cytokines, and together they promote tamoxifen resistance.

### Lnc-DC promotes cytokine production through STAT3

**Fig. 6:**
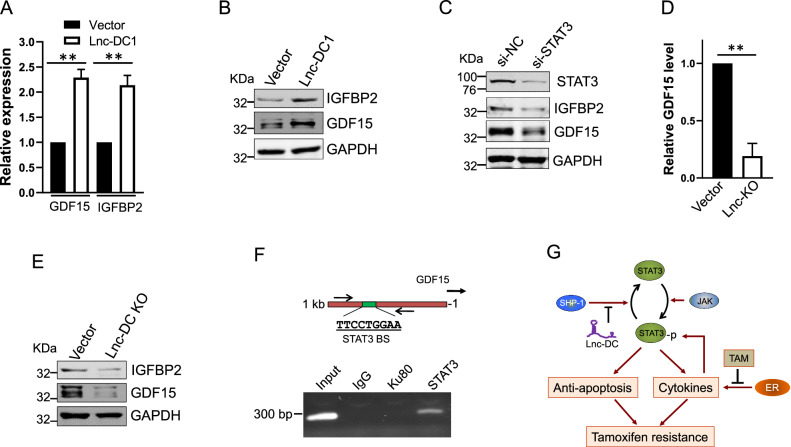
Lnc-DC promotes expression of cytokine genes. **A** Lnc-DC1 upregulates GDF15 and IGFBP2 mRNA level, as detected by qRT-PCR. **B** Lnc-DC1 upregulates GDF15 and IGFBP2 protein level, as detected by western blot. **C** STAT3 siRNA reduces levels of cleaved PARP and Caspase 3 in Lnc-DC1 cells. **D** Lnc-DC KO causes downregulation of GDF15 as determined by qRT-PCR. **E** Lnc-DC KO reduces GDF15 and IGFBP2 protein level, as detected by western blot. **F** STAT3 binds to GDF15 promoter, as detected by ChIP assay. Up panel, schematic description of GDF15 promoter carrying a putative STAT3 binding site. **G** Possible role of Lnc-DC in regulation of STAT3-cytokine loop, leading to tamoxifen resistance. Values are mean ± SD (*n* = 3). **p < 0.01.

To determine the role of Lnc-DC in the STAT3-mediated cytokine production, we also performed cytokine array for the conditioned medium derived from Lnc-DC1 cells or vector control. We identified upregulation of 3 cytokines (GDF15, IGFBP2, and ITF) (Fig. S7). We confirmed that two of three cytokine genes were transcriptionally activated in Lnc-DC1 cells by qRT-PCR (Fig. 6A). We also found increased protein levels of these two cytokines (Fig. 6B). Furthermore, we knocked down STAT3 by siRNA in the Lnc-DC cells and found that STAT3 KD was able to suppress GDF15 and IGFBP2 (Fig. 6C). In addition, Lnc-DC KO downregulated the GDF15 mRNA level (Fig. 6D) as well as protein level (Fig. 6E). Since tamoxifen is the antagonist of ER function, it blocks the transcription of ER-regulated downstream genes. We sought to determine which of these secreted cytokines are regulated by ER, and found that GDF15, IGFBP2, and CXCL12 were down regulated in ER knockdown cells (Fig. S8) based on the GSE7473 GEO database, suggesting that these three genes are directly regulated by ER. In this regard, both GDF15 and IGFBP2 are regulated by ER and STAT3 (Fig. S9), and in particular, we found that GDF15 carries a canonical STAT3 binding site at ~600 bp upstream of GDF15 transcription start site (Fig. 6F, top). ChIP analysis with STAT3 antibody confirmed that STAT3 interacted with GDF15 promoter (Fig. 6F, bottom). A unique PCR band with PCR primers targeting GDF 15 promotor was detected only in STAT3 antibody lane, but not IgG or Ku80 antibody (a negative control) lane. Together, these results suggest that there is a STAT3-cytokine loop where Lnc-DC plays a critical role. As a result, these cells become independent of estrogen, and resistant to tamoxifen (Fig. 6G).

## Discussion

As master gene regulators, lncRNAs have been implicated in regulation of diverse cellular function and disease processes. The present study demonstrates Lnc-DC as a potential lncRNA involved in estrogen independent growth and tamoxifen resistance. Further characterization provides supporting evidence that Lnc-DC is a key regulator for STAT3-cytokine feedback loop, leading to tamoxifen resistance. Dysregulation of Lnc-DC may contribute to the clinical endocrine therapy resistance, and thus, Lnc-DC may serve as a biomarker to predict the response to tamoxifen.

ER is not only a good diagnostic marker for breast cancer, but also serves a therapeutic target. Tamoxifen is one of the important SERMs for ER positive breast cancers [36] and it competitively inhibits the recruitment of transcription coactivators by ER, hence shutting down the transcription of ER responsive genes [37]. However, a critical challenge is the development of resistance. In particular, there is a lack of reliable markers that can predict the response to tamoxifen resistance. Although a meta-analysis study suggests that three protein-coding genes, PGR, MAPT, and SLC7A5, are promising prognostic biomarkers in tamoxifen treated patients [38], it remains to be seen if they can be used for clinical practice. It is evident that additional markers are needed to enhance the accuracy of prediction. The present study suggests that Lnc-DC is not only a potential prognostic marker for overall patient survival, but it is able to predict tamoxifen therapy.

Mechanisms of tamoxifen resistance are complex and may involve multiple factors such as AKT, HER2, and R-Ras [39–42]. In addition, a study has implicated MACROD2 in estrogen independent growth and tamoxifen resistance by enhancing p300 binding to estrogen response elements in a subset of ER regulated genes [43]. However, these studies have been focusing on protein-coding genes. On the other hand, much less is known about the role of lncRNAs in tamoxifen resistance although the number of lncRNAs is much larger than that of protein coding genes [44]. Thus, our study provides new insight into the lncRNA-mediated tamoxifen resistance through Lnc-DC/STAT3/cytokine axis, highlighting a cross talk between STAT3 activation and cytokines where Lnc-DC serves as a master regulator.

Lnc-DC was first reported to be required for the differentiation of monocytes into dendritic cells by regulating STAT3 phosphorylation and it appears to be expressed exclusively in dendritic cells [31]. Lnc-DC may serve as a decoy to block the dephosphorylation of STAT3 by SHP-1 [31]. The transcription of Lnc-DC is under control of Pu.1, an ETS-domain transcription factor that activates gene expression during myeloid and B-lymphoid cell development [45]. However, little is known regarding the function of Lnc-DC beyond dendritic cells. We show that Lnc-DC is expressed in breast cancer cells. Furthermore, similar to what has been reported in dendritic cells, Lnc-DC is also able to regulate phosphorylation of STAT3. For example, activation of Lnc-DC by SAM gRNA causes upregulation of pSTAT3^Y705^, whereas Lnc-DC KO suppresses pSTAT3^Y705^ level. We further show that the interaction of Lnc-DC with STAT3 reduces the binding of SHP-1 to STAT3. In addition, Lnc-DC is able to stimulate the secretion of the cytokines such as GDF15 and IGFBP2. Finally, these Lnc-DC 1 expressing cells also reveal a high level of GDF15 and IGFBP2.

STAT3 becomes activated through phosphorylation on tyrosine residue as a DNA binding protein in response to epidermal growth factor and interleukin-6 [46]. Thus, STAT3 is a major cell survival pathway, and it controls different downstream genes [47]. Accumulating evidence indicates that STAT3 plays a critical role in cancer signaling through inflammation, obesity, stem cells, and the pre-metastatic niche [48]. In addition, STAT3 regulates mitochondrion functions, as well as gene expression through epigenetic mechanisms [48]. STAT3 has also been implicated in therapy resistance [49]. Similarly, STAT3 has been implicated in herceptin-induced resistance in HER2-overexpressing breast tumors [50]. Moreover, there is a reciprocal regulation of pSTAT3 and RANTEN via an autocrine loop, contributing to tamoxifen resistance [32]. In consistence with these findings, our results suggest that multiple cytokines are involved in maintaining this STAT3-mediated autocrine loop.

The gain of function screening can facilitate identification of new gene function important to different human diseases. Our CRISPR/Cas9-based SAM library screen provides such an example for identification of novel function of lncRNAs. Since overwhelming number of lncRNAs are identified to date, it is difficult to include every one of them in a single library. To tackle this challenge, we focus on a group of lncRNAs which are selected based on literature information. These lncRNAs are associated with physiological function or their aberrant expression is associated with certain disease conditions. Although such a library may miss some important hits, an advantage of the small number of lncRNAs in this library would allow us to design multiple gRNAs per lncRNA to enhance the change of gene activation. This design may be critical for functional screen because not every gRNA is equally able to activate the corresponding lncRNA. For instance, we found that only one of five Lnc-DC SAM gRNAs induces a high level of the endogenous Lnc-DC expression.

In summary, we demonstrate a critical role for Lnc-DC in regulation of STAT3-cytokine loop, leading to estrogen independency and tamoxifen resistance. Furthermore, clinical data mining suggests that Lnc-DC may serve as a potential marker for prediction of tamoxifen response. In this regulatory system, JAK promotes STAT3 phosphorylation, whereas SHP-1 promotes dephosphorylation. The interaction of Lnc-DC with STAT3 may prevent SHP-1’s action on pSTAT3, keeping a high level of pSTAT3. STAT3 is known to be capable of upregulating anti-apoptotic genes such as Bcl2 and stimulating cytokine production. Since cytokines such as CXCL12, IGFBP2 and GDF15 can be regulated by both ER and STAT3, it is conceivable that these cytokines, in addition to anti-apoptotic genes, can be kept producing in the elevated Lnc-DC background such that these tumor cells can survive and grow even when ER signaling is shut down by clinical intervention such as estrogen deprivation or tamoxifen treatment (Fig. 6D). Given that lncRNAs often play multiple functions, it would be interesting to determine whether Lnc-DC can regulate cytokine production independent of STAT3. On the other hand, further characterization of tamoxifen-mediated regulation of Lnc-DC may provide new insight into clinical tamoxifen resistance.

## Supplementary information

Supplementary figures

Supplementary table 1

Supplementary table 2

## Data Availability

All data generated in this study are included either in this article or in the Supplementary Information files.
