# Correction: Lnc-DC promotes estrogen independent growth and tamoxifen resistance in breast cancer

**DOI:** 10.1038/s41419-024-07301-5

**Published:** 2025-01-13

**Authors:** Wan-Xin Peng, Pratirodh Koirala, Huaixiang Zhou, Jiahong Jiang, Ziqiang Zhang, Liu Yang, Yin-Yuan Mo

**Affiliations:** 1 Center of Oncology, Department of Medical Oncology, Zhejiang Provincial People’s Hospital, People’s Hospital of Hangzhou Medical College, Hangzhou, Zhejiang PR China; 2 Cancer Institute, University of Mississippi Medical Center, Jackson, MS USA; 3 Department of Biochemistry, University of Mississippi Medical Center, Jackson, MS USA; 4 Department of Pulmonary Medicine, Tongji Hospital, Tongji University, Shanghai, China; 5 Department of Pharmacology/Toxicology, University of Mississippi Medical Center, Jackson, MS USA

**Keywords:** Breast cancer, Non-coding RNAs

Correction to: *Cell Death & Disease* 10.1038/s41419-021-04288-1, published online 25 October 2021

In this article Fig. 5F has been given erroneously.
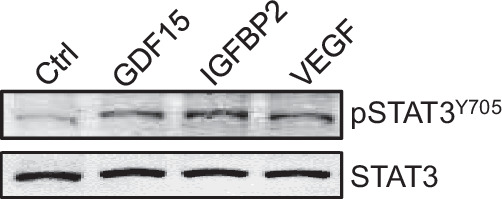


We realized that when we composed figures, we put in a wrong picture due to several people involved in this work.

The original article has been corrected.

