# Supplementary material for: Lnc-DC promotes estrogen independent growth and tamoxifen resistance in breast cancer
Source: Cell Death Dis. 2021 Oct 25;12(11):1000. doi: 10.1038/s41419-021-04288-1 (PMC8546148; doi:10.1038/s41419-021-04288-1)
Supplement: Supplementary file 1 — Supplementary figures [file 41419_2021_4288_MOESM1_ESM.pdf]

Supplementary Figures

Fig. S1

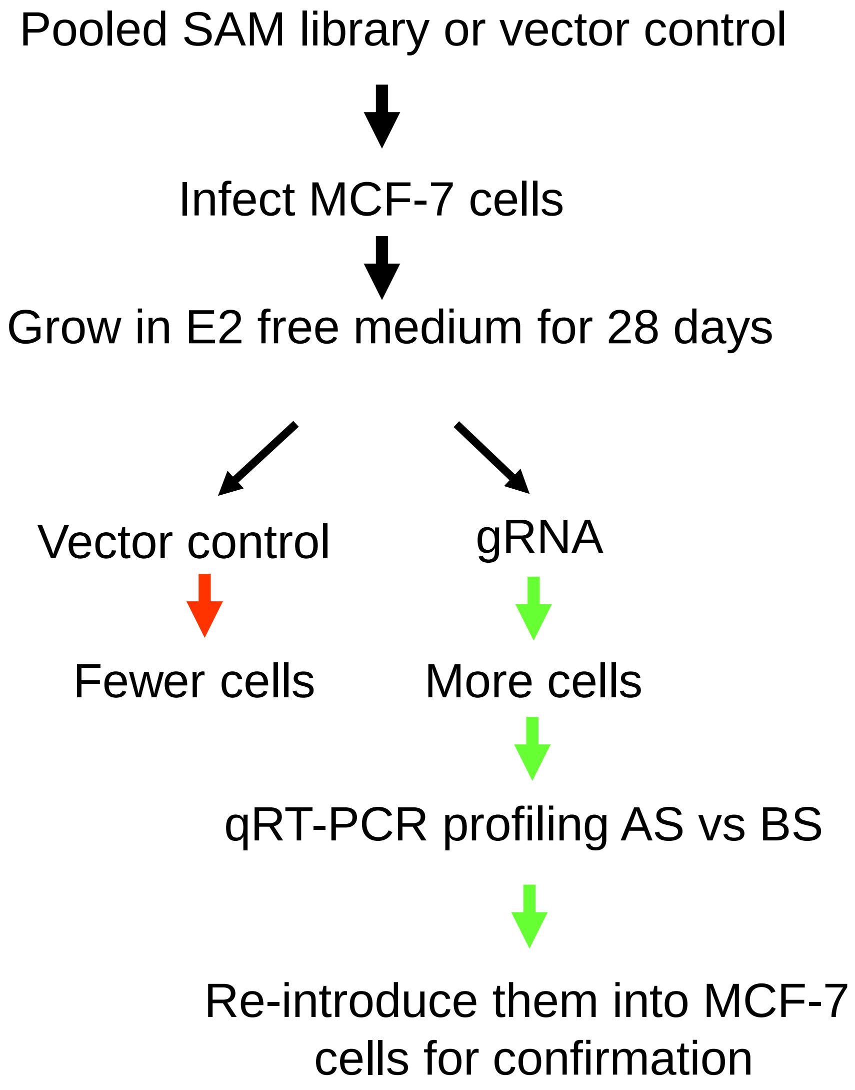
Pooled SAM library or vector control
Infect MCF-7 cells
Grow in E2 free medium for 28 days
Vector control
gRNA
Fewer cells
More cells
qRT-PCR profiling AS vs BS
Re-introduce them into MCF-7 cells for confirmation

Fig. S2

A

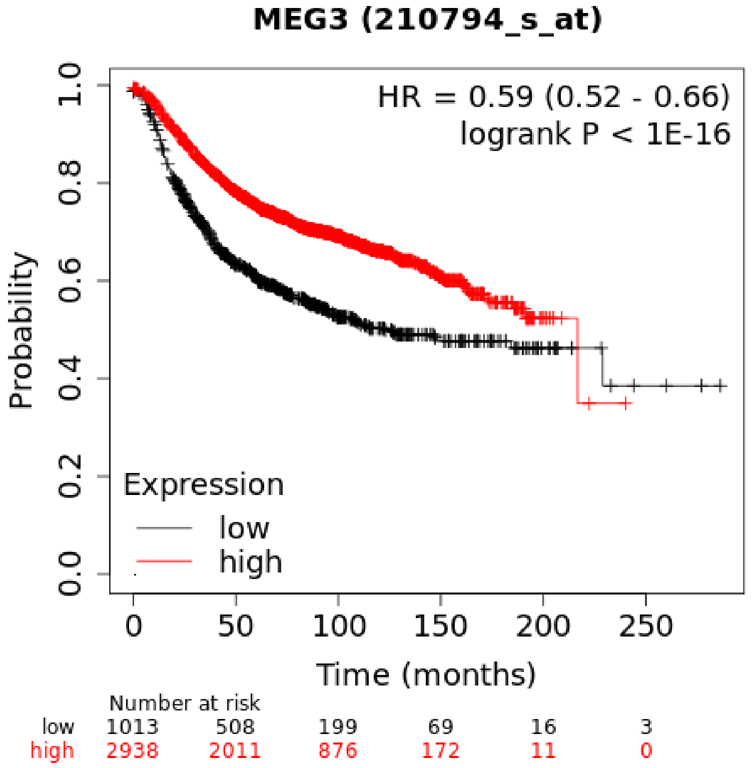
MEG3 (210794_s_at)
HR = 0.59 (0.52 - 0.66)
logrank P < 1E-16
Probability
Expression
low
high
Time (months)
Number at risk
low 1013 508 199 69 16 3
high 2938 2011 876 172 11 0

B

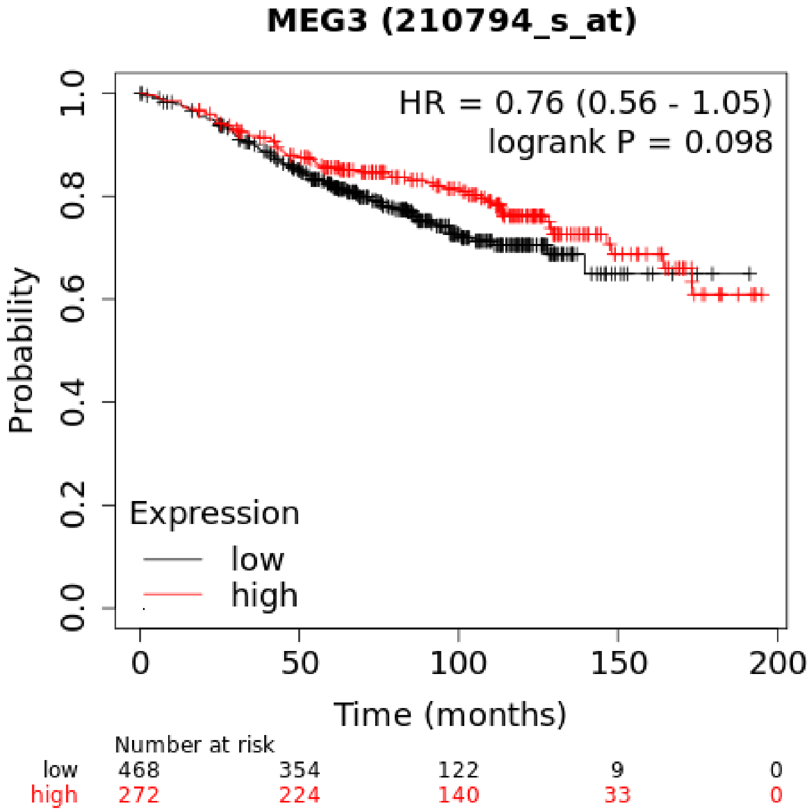
MEG3 (210794_s_at)
HR = 0.76 (0.56 - 1.05)
logrank P = 0.098
Probability
Expression
low
high
Time (months)
Number at risk
low 468 354 122 9 0
high 272 224 140 33 0

Fig. S3

Early relapse group (DFS < 36.39 months)

Patient ID	Relapse time	Lnc-DC expression
1235	22.26	6.44556
1240	25.39	6.09208
1253	25.16	6.00899
1256	24.98	6.31805
1257	27.46	6.3208
1260	25.42	6.15003
1262	25.76	6.95216
1267	27.3	6.22161
1272	26.15	6.09939
1276	25.03	5.80016
1281	25.36	6.95249

Late relapse group (DFS > 36.39 months)

Patient ID	Relapse time	Lnc-DC expression
1237	62.16	6.42473
1243	60.32	5.99213
1250	55.88	5.51257
1254	50.49	5.95856
1265	47.7	6.25525
1271	39.35	5.73554
1277	40.67	5.99925
1282	41.06	5.76517
1284	60.15	5.99511
1286	56.24	6.14866

Fig. S4

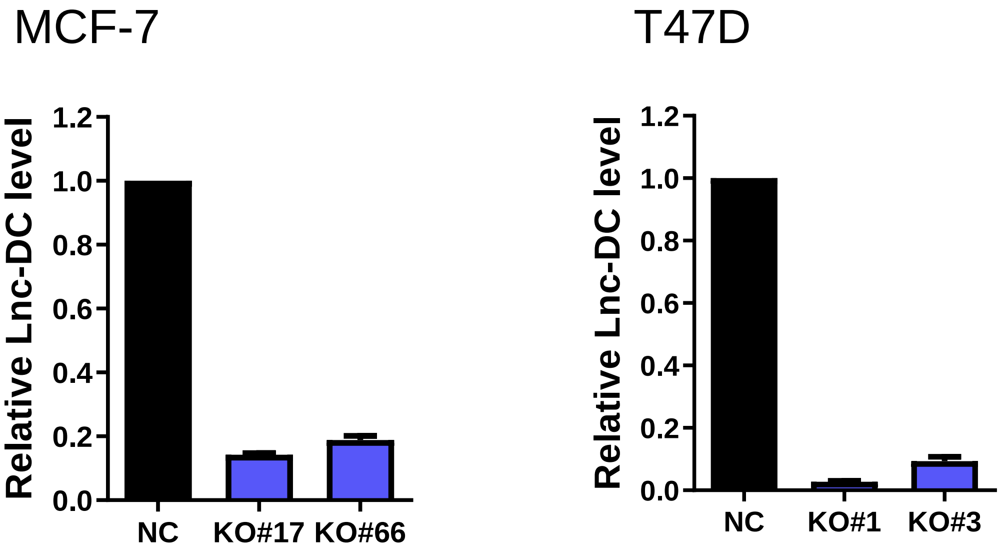

MCF-7
Relative Lnc-DC level
1.2
1.0
0.8
0.6
0.4
0.2
0.0
NC
KO#17
KO#66
T47D
Relative Lnc-DC level
1.2
1.0
0.8
0.6
0.4
0.2
0.0
NC
KO#1
KO#3

Fig. S5

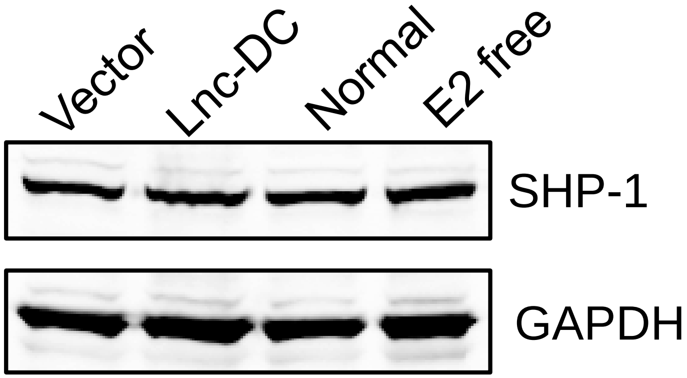

Fig. S6

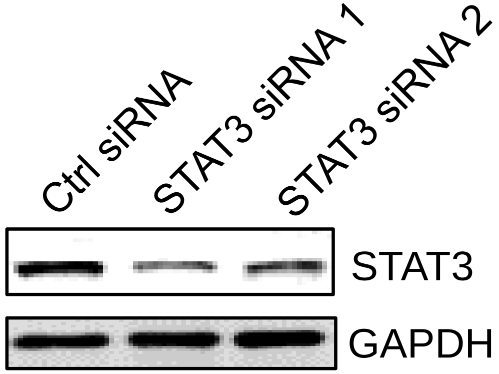

Fig. S7

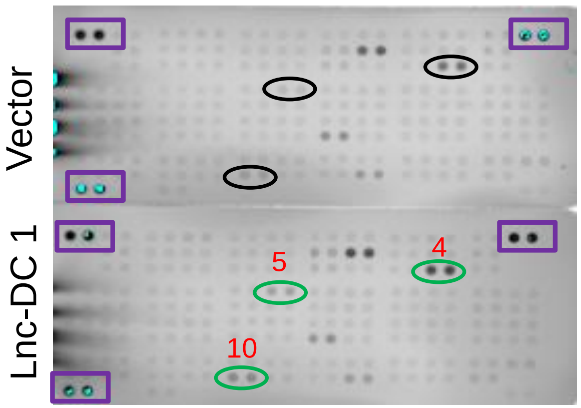

4	GDF15
5	IGFBP2
10	ITF

Fig. S8

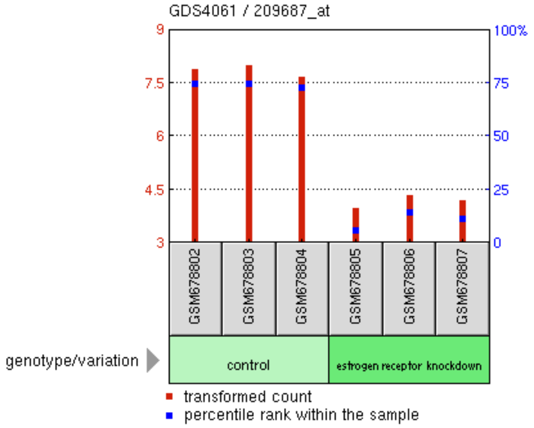

CXCL12

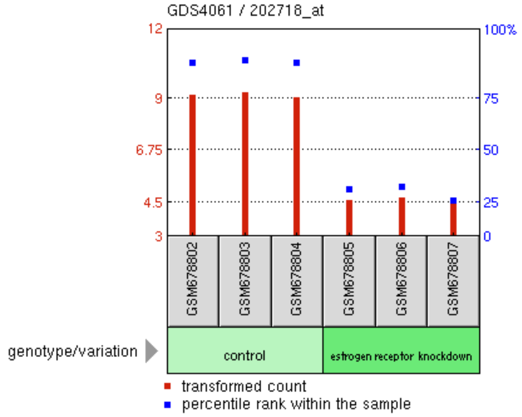

IGFBP2

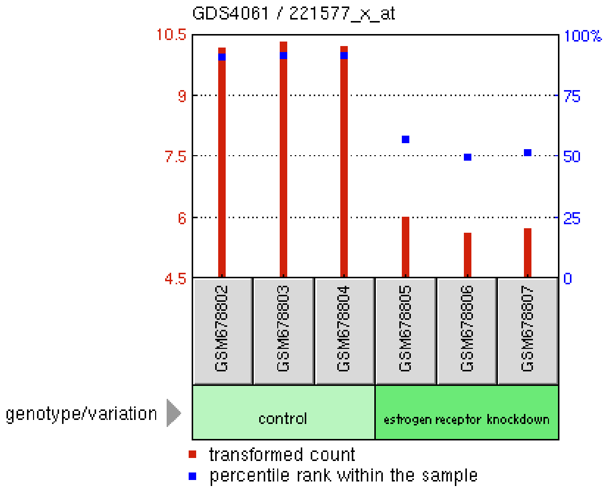

GDF15

Fig. S9

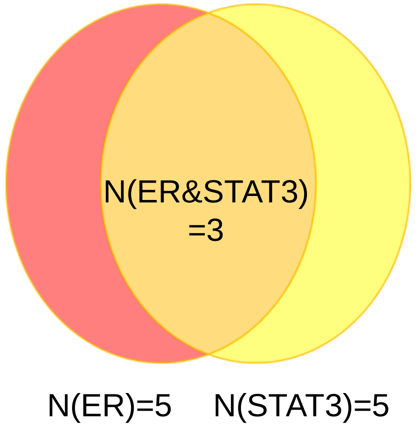

Gene	STAT3 Kd	ER Kd
CXCL12	↓	↓
GDF15	↓	↓
IGFBP2	↓	↓
PDGF-A	↓	-
VEGF	↓	-

Supplementary figure legend

Fig. S1 Strategy for identification of lncRNAs involved in estrogen independent growth. AS, after selection; BS, before selection.

Fig. S2 A, Low expression of MEG3 is associated with poor relapse free survival (RFS) in 3951 cases, using the kmplot program (www.kmplot.com) with auto select best cutoff setting. B, MEG3 express is not associated with the response to tamoxifen treatment (740 cases).

Fig. S3 Lnc-DC expression and relapse time based on GSE16391.

Fig. S4 Detection of Lnc-DC expression in MCF-7 and T47D Lnc-DC KO cells by qRT-PCR.

Fig. S5 Lnc-DC or estrogen deprivation has no effect on SHP-1 expression. For Lnc-DC overexpression, MCF-7 cells were infected with lentivirus and cellular extract was prepared 2 days after infection. For estrogen deprivation, MCF-7 cells were cultured in E2 free medium for one week before harvesting for western blot.

Fig. S6 Suppression of STAT3 by RNAi in MCF-7 cells as detected by Western blot.

Fig. S7 Detection of higher levels of three cytokines in conditioned medium from Lnc-DC1 expressing cell culture than in vector control. Dots in purple boxes are loading controls.

Fig. S8 Expression of transcript in ERα knockdown MCF-7 cells (GSE27473). The selected genes (CXCL12, IGFBP2 and GDF15) were downregulated.

Fig. S9 Venn diagram reveals that three of five cytokine genes, CXCL12, GDF15 and IGFBP2, are controlled by both ER and STAT3, based on the GSE7473 GEO database.
