# Supplementary material for: Lnc-DC promotes estrogen independent growth and tamoxifen resistance in breast cancer
Source: Cell Death Dis. 2021 Oct 25;12(11):1000. doi: 10.1038/s41419-021-04288-1 (PMC8546148; doi:10.1038/s41419-021-04288-1)
Supplement: Supplementary file 2 — Supplementary table 1 [file 41419_2021_4288_MOESM2_ESM.pdf]

Supplemental Tables

Supplemental Table 1, Primers and gBlock fragment used in this study

Supplemental Table 2, gRNA sequences in the SAM library

Supplemental Table 1, Primers and gBlock fragment used in this study

Lnc-DC-RT-5.1	ccttccaacagcaacatgaa
Lnc-DC-RT-3.1	tgtccttaccctgcaacaca
Lnc-DC-S1m-R1-5.1	attctagagctagcgaattcGTGTTTTTCCAAAGCCTTCCA
Lnc-DC-S1m-BamH1-3.1	TtttctacggatccggatccGCTTTTAACAATGATGATTTAT
Cpf1-LNC-DC-T1a	tgtcagaggagggcgttcccttg
Cpf1-LNC-DC-T1b	tgtcctactcatttctggaaagt
Lnc-DC1-SAM-1-5.1	CACCgAGGACCCAGGGTACACAGGG
Lnc-DC1-SAM-1-3.1	aaacCCCTGTGTACCCTGGGTCCTc
Lnc-DC1-SAM-2-5.1	CACCgGAGCTGGAATGCAAGGCCAG
Lnc-DC1-SAM-2-3.1	aaacCTGGCCTTGCATTCCAGCTCc
Lnc-DC1-SAM-3-5.1	CACCgGCTGTGAGTGACTGAGAAAA
Lnc-DC1-SAM-3-3.1	aaacTTTTCTCAGTCACTCACAGCc
Lnc-DC1-SAM-4-5.1	CACCgGTCAGAGGAGGGCGTTCCCT
Lnc-DC1-SAM-4-3.1	aaacAGGGAACGCCCTCCTCTGACc
Lnc-DC1-SAM-5-5.1	CACCgAGGTAATGTAGAAGGACCCA
Lnc-DC1-SAM-5-3.1	aaacTGGGTCCTTCTACATTACCTc
Lnc-DC sgRNA1 5.1	aggacccagggtacacaggg
Lnc-DC sgRNA2 5.1	gagctggaatgcaaggccag
Lnc-DC sgRNA3 5.1	gctgtgagtgactgagaaaa
Lnc-DC sgRNA4 5.1	gtcagaggagggcgttccct
Lnc-DC sgRNA5 5.1	aggtaatgtagaaggaccca
Lnc-DC sgRNA1 3.1	aaactgggtccttctacattacctc
SAM gRNA-5.1	tttcttggctttatatatcttGTGGAAAGGACGAAACACC
SAM gRNA-3.1	CATGTTggccaagttgataacggactagccttattttaac
VEGF165-5.1	ccctgatgagatcgagtacatctt
VEGF165-3.1	agcaaggcccacagggattt
GDF15-5.1	tcagagttgcactccgaaga
GDF15-3.1	agagatacgcaggtgcaggt
IGFBP2-5.1	gagaaggtcactgagcagca
IGFBP2-3.1	gggatgtgcagggagtagag
CXCL12-5.1	tgagagctcgctttgagtga
CXCL12-3.1	ggaaatgctaccttgccaac
PDGFA-5.1	acgtcaggaagaagccaaaa
PDGFA-3.1	ggctcatcctcacctcacat
GDF15-ChIP-5.1	TCCTATGTGTCTGGCCCTGT
GDF15-ChIP-3.1	CTCTTTTTGGTTGGGGTCAA
GDF15-ChIP-5.2	GGAGCACCCTGCTTAGACTG
GDF15-ChIP-3.2	TCTCACTGGAGAGGTGGTCA

MS2-Nhe-BamH-gBlock

AGATCCAGTTTGGTTAATTA**GCTAGC**GAGGGCCTATTTCCCATGATTCCTTCATATTTGCATATACGATACAAGGCT
GTTAGAGAGATAATTGGAATTAATTTGACTGTAAACACAAAGATATTAGTACAAAATACGTGACGTAGAAAGTAATA

ATTTCTTGGGTAGTTTGCAGTTTTAAAATTATGTTTTAAAATGGACTATCATATGCTTACCGTAACTTGAAAGTATT
TCGATTTCTTGGCTTTATATATCTTGAGACGGTGGAAAGGACGAAACACCGGGATACTGTTTTAGAGCTAGGCCAAC
ATGAGGATCACCCATGTCTGCAGGGCCTAGCAAGTTAAAATAAGGCTAGTCCCGTCTCGTTATCAACTTGGCCAACA
TGAGGATCACCCATGTCTGCAGGGCCAAGTGGCACCGAGTCGGTGCTTTTTTT**GGATCC**TGCAAAGATGGATAAAGT
TT
